# Nomograms based on ratio indexes to predict severity and prognosis in immune checkpoint inhibitors-related myocarditis: a retrospective analysis

**DOI:** 10.1007/s00432-024-05801-7

**Published:** 2024-05-27

**Authors:** Zhenli Li, Tiezhu Yao, Guang Liu, Zhengkun Guan, Jing Liu, Ling Guo, Jingtao Ma

**Affiliations:** Department of Cardiology, The Fourth Hospital of Hebei Medical University, 12 Jiankang Road, Shijiazhuang, 050011 Hebei Provence People’s Republic of China

**Keywords:** ICI-associated myocarditis, MACE, Predictive model, Prognostic analysis, Diagnostic analysis, Nomogram

## Abstract

**Purpose:**

Immune checkpoint inhibitors-related myocarditis (ICI-M) is one of the immune-related adverse events (irAEs), which is rare and highly lethal. This study aimed to establish nomograms based on ratio biomarkers to predict the severity and prognosis of ICI-M.

**Methods:**

We retrospectively examined patients with advanced cancers who were also diagnosed with ICI-M at the Fourth Hospital of Hebei Medical University. The patients of ICI-M were divided into mild and severe groups and a 40-day following up was carried out. The major adverse cardiovascular events(MACEs) were regarded as the endpoint. Nomogram-based models were established and validated.

**Results:**

Seventy-seven patients were involved, including 31 severe cases(40.3%). Lactate dehydrogenase-to-albumin ratio(LAR) combined with the change rate from baseline to onset of LAR($\triangle$LAR) which performed best to diagnose the severe ICI-M was identified to establish the nomogram-based model. The bootstrap-corrected concordance index [0.752 95% confidence interval (CI): 0.635$-$0.866] and calibration plot with good degree of fitting confirmed this diagnostic model. Neutrophil-to-high-density lipoprotein cholesterol ratio(NHR) and LAR were also screened into the nomogram-based model for 40-day MACEs after ICI-M, which performed well by validating for concordance index(0.779 95% CI: 0.677$-$0.865)and calibration plots after being bootstrap-corrected. Moreover, a $\ge$101% increase in LAR significantly separated patients in MACE-free survival.

**Conclusion:**

Ratio indexes at onset and their change rates from baseline showed good diagnostic value for the severity of ICI-M and prognostic value for subsequent MACEs, particularly LAR, NHR and their change rates. The nomogram-based models of ratio indexes could provide a potential choice for early detection and monitor of the severe ICI-M and subsequent MACEs.

**Graphical abstract:**

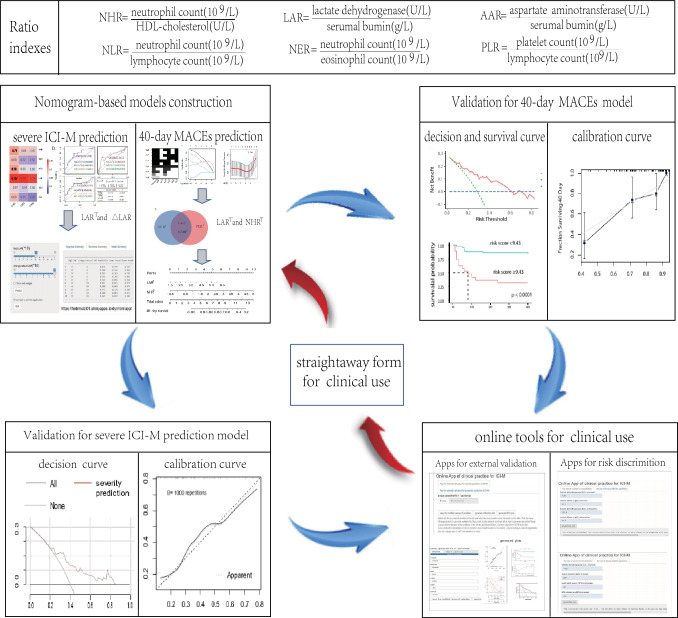

**Supplementary Information:**

The online version contains supplementary material available at 10.1007/s00432-024-05801-7.

## Introduction

Immune checkpoint inhibitors (ICIs) emerged as a novel tumor therapy, which have significantly improved prognosis in patients with advanced metastatic cancers (e.g., melanoma, non-small cell lung cancer, renal cell carcinoma) (Vergara et al. 2023). ICIs amplify T-cell-mediated immune response against cancer cells by blocking intrinsic down-regulators, which include cytotoxic T-lymphocyte antigen 4 (CTLA-4)and programmed cell death 1 (PD-1) or its ligand of programmed cell death ligand 1 (PD-L1) (Moslehi et al. 2021; Jo et al. 2024).

The systemic immune hyperreactivity induced by ICIs may lead to immune-related adverse events (irAEs), which rechallenge the effectiveness and safety of such therapy strategy (Yousif et al. 2023). However, the mechanism of irAEs is still unclear, though some researches have demonstrated that the activation of T cells and deleterious effects of elevated proinflammatory cytokines from activated immune cells may play an important role (Baik et al. 2021; Liu et al. 2024). Immune checkpoint inhibitors-related myocarditis (ICI-M), as one of the most lethal irAEs, is pathologically characterized by macrophage and T cell infiltration with a incidence ranging from 0.27 to 1.14% (probably underestimated due to asymptomatic confusion)and a mortality up to 50%; it occurs early with a median time of from 18 to 60 days after 1 or 2 doses of immunotherapy (Hu et al. 2019). Therefore, there exists a critical need for the early monitoring, diagnosis and management of ICI-M. A higher grade of ICI-M usually means a parallelly increased intensive care requirement and mortality, which highlights the necessity of the severity assessment of it. Besides, the major adverse cardiovascular events (MACEs) following ICI-M are even more lethal, but the diagnostic algorithm and risk stratification for cardiological sequelae are still debated (Vergara et al. 2023).

In recent years, a series of ratio indexes derived from circulating biomarkers have been explored as predictors of treatment response and clinical outcomes of ICI therapy. In some studies, increased neutrophil-to-lymphocyte ratio (NLR) showed more ICI-associated cardiovascular toxicity in patients with pre-existing cardiovascular diseases (Haj-Yehia et al. 2024). An association between increased neutrophil-to-eosinophil ratio (NER) at ICI-related cardiotoxicities (iRCs) onset and the development of severe iRCs was discovered (Liang et al. 2023). A group of promising biomarkers, including LDH, lactate dehydrogenase-to-albumin ratio (LAR), troponin I, and aspartate aminotransferase-to-albumin(AAR), excellently predicted the 30-day mortality rate of ICI-M (Zhuang et al. 2024). In other studies, time-dependent changes of NLR and platelet-to-lymphocyte ratio (PLR) were also used to identify irAEs and assess cardiovascular risks (Vergara et al. 2023; Takada et al. 2022). However, the relationship between ICI-M and the change rates of ratio indexes have not been fully clarified. Moreover, clinical prediction models for the diagnosis and prognosis of ICI-M have not been exploit currently. This study aimed to fully exploit the predictive value of ratio indexes as well as their change rates for the severity of ICI-M and the MACEs after ICI-M and establish potential models for clinical use.

## Methods

### Study population

The electronic medical records of patients with a pathologic diagnosis of advanced cancers who received at least one cycle of ICI therapy at the Fourth Hospital of Hebei Medical University from January 2020 to December 2023 were reviewed. Inclusion criteria were adequate follow-up, and treatment with ICIs according to clinical practice. Cases lost to follow-up and cases without ICI therapy according to instructions were excluded. A total of 77 patients who had been diagnosed with ICI-M according to the guidelines of the European Society of Cardiology were included (Caforio et al. 2013). All ICI-M cases were confirmed by two cardiovascular specialists based on the existing clinical evidence.

### Data collection

Data were collected retrospectively from electronic medical records including cancer types with stage grades, eastern Cooperative Oncology Group performance status (ECOG PS), past treatments, cardiovascular risk factors, medical comorbidities, detailed immunotherapy-related information, cardiovascular outcomes during the following up, cardiac-related parameters [troponin I (TnI), N-terminal probrain natriuretic peptide (NT-proBNP) and left ventricular ejection fraction (LVEF)] and ratio biomarkers [Neutrophils-to-high-density lipoprotein cholesterol ratio(NHR), NLR, NER, PLR, LAR and AAR (calculated from the complete blood count, biochemical examination, or both)]. Cardiac biomarkers were obtained according to the clinicians’ discretion (at baseline, at onset, or both). Ratio indexes (NHR, NLR, NER, PLR, LAR and AAR) were calculated by two time nodes of baseline (immediately prior to the initial ICI therapy) and onset of ICI-M (upon the suspected diagnose of ICI-M).

### Definitions and outcomes of interest

The laboratory data analyzed in this study was obtained immediately upon admission of a patient to the hospital. All blood parameters were measured using standard methods at the central laboratory of the Fourth Hospital of Hebei Medical University. ICI-M was graded by the Common Terminology Criteria for Adverse Events (CTCAE) criteria version 4.03. CTCAE grades $\le$ 2 were classifed as mild myocarditis, whereas grades $\ge$ 3 were severe myocarditis. We explored a series of emerging ratio indexes and their change rates based on previous reports. The formulas of the ratio indexes are as follows:

$$\begin{aligned} NHR= & {} \frac{\text {neutrophil count } (10^9/L)}{\text {HDL-cholesterol(U/L)}}\\ LAR= & {} \frac{\text {lactate dehydrogenase (U/L)}}{\text {serum albumin(g/L)}}\\ NLR= & {} \frac{\text {neutrophil count}(10^9/L)}{\text {lymphocyte count } (10^9/L)}\\ NER= & {} \frac{\text {neutrophil count } (10^9/L)}{\text {eosinophil count } (10^9/L)} \\ AAR= & {} \frac{\text {aspartate aminotransferase(U/L)}}{\text {serum albumin (g/L)}}\\ PLR= & {} \frac{\text {platelet count } (10^9/L)}{\text {lymphocyte count}(10^9/L)} \end{aligned}$$

To better display and analyse the indexes, we used logarithmic form of indexes in some circumstances, the formula and an example are as follows:

$$\begin{aligned} {\mathrm{Ratio \, index}}^T= \log _2\left( \text {Ratio index}\right) \ \ \ \ \ \ \ \ \ \ (\text {e.g.})\textrm{NHR}^T= \log _2\left( \text {NHR}\right) \end{aligned}$$

The formula of the change rates of ratio indexes and an example are as follows:

$$\begin{aligned} \Delta \text {Ratio index}= & {} \left( \frac{\text {Ratio index}_{\text {onset}} - \text {Ratio index}_{\text {baseline}}}{\text {Ratio index}_{\text {baseline}}}\right) \\{} & {} \times 100\% \\ (e.g.)\Delta \text {NHR}= & {} \left( \frac{\text {NHR}_{\text {onset}} - \text {NHR}_{\text {baseline}}}{\text {NHR}_{\text {baseline}}}\right) \times 100\% \end{aligned}$$

Especially, as for eosinophils of zero, the eosinophils of these patients was adjusted to 0.01 $\times 10^9$ /L to avoid zero in the denominator. The main outcome of interest was the occurrence of MACEs after a 40-day following up, which were defined as cardiovascular death, cardiac arrest, cardiogenic shock, and hemodynamically significant complete heart block that happened after the diagnosis of ICI-M for the first time (Zhang et al. 2020). Time to onset of ICI-M was defined as the number of days from the initial ICI treatment to presentation of ICI-M. Time to MACEs was defined as the number of days from the diagnosis of ICI-M to presentation of the earliest MACE. The earliest event was defined as the first MACE in patients with multiple MACEs.

### Statistical analysis

Numbers and proportions (%) were used to present categorical variables. Continue variables were presented as the mean with standard deviation (SD) or the median with interquartile range (IQR) according to whether a variable had a normal distribution, where Shapiro-Wilk tests were used. T test, paired Wilcoxon signed-rank test and Wilcoxon signed-rank were used to compare continuous variables and Chi-square or Fisher’s exact test for categorical variables according to the particular situation. As for the comparison of ratio indexes, indexes were displayed in the logarithmic form, but the initial indexes were used to compare them by appropriate methods. Univariate and multivariate logistic and cox regression were performed for screening each potential risk variable. Best subset regression (BSR) analysis and least absolute shrinkage and selection operator (LASSO) regression analysis were used to select the best relative indexes to predict the MACEs. Area under curves (AUC) with 95% confidence interval (CI) of the receiver operating characteristic (ROC) analysis were used to compare the diagnostic ability of risk scores from different logistic regression models with single indexes. Spearman correlation test was also used to show the correlation between 2 variables. Survival curves were visualized by the Kaplan-Meier analysis and log-rank analysis after dividing ICI-M patients into low and high groups using median value of different indexes. Nomograms of logistic regression and Cox regression were established and validated by the concordance index (C-index) and calibration plots using bootstrap. Decision curve analyses (DCA) were also performed. All statistical analyses were performed using R software (4.3.2). Probability values of *p*<0.05 indicated a statistically significant difference.

## Results

### Characteristics of patients

As displayed in Table 1, a total of 77 patients of ICI-M including 54 male patients (70.13%)and 23 female patients (29.87%) with a median age of 67 years (63–71 years) were involved in this study. As for camorbidities, only diabetes showed significant difference between the mild (32.61%) and severe (58.06%) groups. Among patients predominantly composed of gastrointestinal tumors (62.34%), prior cancer treatments did not differ between patients in mild and severe groups, as was the same to ECOG PS and ICI therapy cycles. The median interval from treatments to ICI-M and from ICI-M to MACEs was respectively 48 days (28–86 days) and 6 days (3$-$8.5 days), but both displayed no significant difference when divided by the severity of ICI-M. However, patients with severe ICI-M were more likely to develop irAEs (32.96% vs. 61.29%;*p*=0.036). The circulating parameters, ratio indexes and cardiac-related parameters are displayed in in Table S1. Totally, 73 (94.8%) and 3 (3.9%) patients received PD-1 and PD-L1 inhibitors therapy respectively, except one case received a PD-1/CTLA-4 inhibitor. The detailed administration of ICIs in different tumors, exact prior combined treatments and the composition of irAEs can be found in Fig. S2, in which the occurrence number was displayed.

**Table 1:** Baseline characteristics

Characteristics	Total (N=77)	Severity of ICI-M		P value
		Mild group (N=46)	Severe group (N=31)	
*Basic information*				
Sex				
Female	23 (29.87%)	13 (28.26%)	10 (32.26%)	Ref
Male	54 (70.13%)	33 (71.74%)	21 (67.74%)	0.707
Age				
Median(IQR)	67 (63, 71)	67 (63, 72)	66 (62, 71)	0.815
BMI	$23.5 \pm 3.3$	$23.3 \pm 3.2$	$23.8 \pm 3.5$	0.581
Smoking history				
No	45 (58.44%)	26 (56.52%)	19 (61.29%)	Ref
Yes	32 (41.56%)	20 (43.48%)	12 (38.71%)	0.677
Comorbidities				
Hypertension	34 (44.16%)	18 (39.13%)	16 (51.61%)	0.279
Diabetes mellitus	33 (42.86%)	15 (32.61%)	18 (58.06%)	0.027
Hyperlipidemia	21 (27.27%)	15 (32.61%)	6 (19.35%)	0.200
Pre-existing				
Cardiovascular disease	32 (41.56%)	15 (32.61%)	17 (54.84%)	0.052
Stroke	16 (20.78%)	10 (21.74%)	6 (19.35%)	0.800
*Tumor-related information*				
Tumor type				
Others	29 (37.66%)	18 (39.13%)	11 (35.48%)	Ref
Gastrointestinal tumors	48 (62.34%)	28 (60.87%)	20 (64.52%)	0.746
Tumor stage				
III	26 (33.77%)	17 (36.96%)	9 (29.03%)	
IV	51 (66.23%)	29 (63.04%)	22 (70.97%)	0.471
ECOG PS				
0–1	51 (66.23%)	31 (67.39%)	20 (64.52%)	Ref
$\ge 2$	26 (33.77%)	15 (32.61%)	11 (35.48%)	0.794
*Immunotherapy-related information*				
Immunotherapy types				
Others	4 (5.19%)	2 (4.35%)	2 (6.45%)	Ref
PD-1	73 (94.81%)	44 (95.65%)	29 (93.55%)	$>0.999$
Prior therapy				
Chemotherapy	60 (77.92%)	35 (76.09%)	25 (80.65%)	0.636
Targeted therapy	21 (27.27%)	12 (26.09%)	9 (29.03%)	0.776
Radiotherapy	15 (19.48%)	8 (17.39%)	7 (22.58%)	0.573
Surgery	18 (23.38%)	14 (30.43%)	4 (12.90%)	0.075
Therapeutic cycles				
Median (IQR)	2.00 (1.0,3.0)	2.0 (1.0,3.0)	2.0 (1.0,3.5)	0.857
Days to ICI-M				
Median (IQR)	48 (28, 98)	48 (31, 98)	38 (28, 87)	0.852
Days to MACEs				
Median (IQR)	6 (3, 8.5)	6.0 (2.5, 10.0)	5 (2.5, 7.5)	0.911
irAEs				
No	41 (53.25%)	29 (63.04%)	12 (38.71%)	Ref
Yes	36 (46.75%)	17 (36.96%)	19 (61.29%)	0.036

### The combination of LAR and $\Delta$LAR to predict the severity of ICI-M

**Fig. 1:**
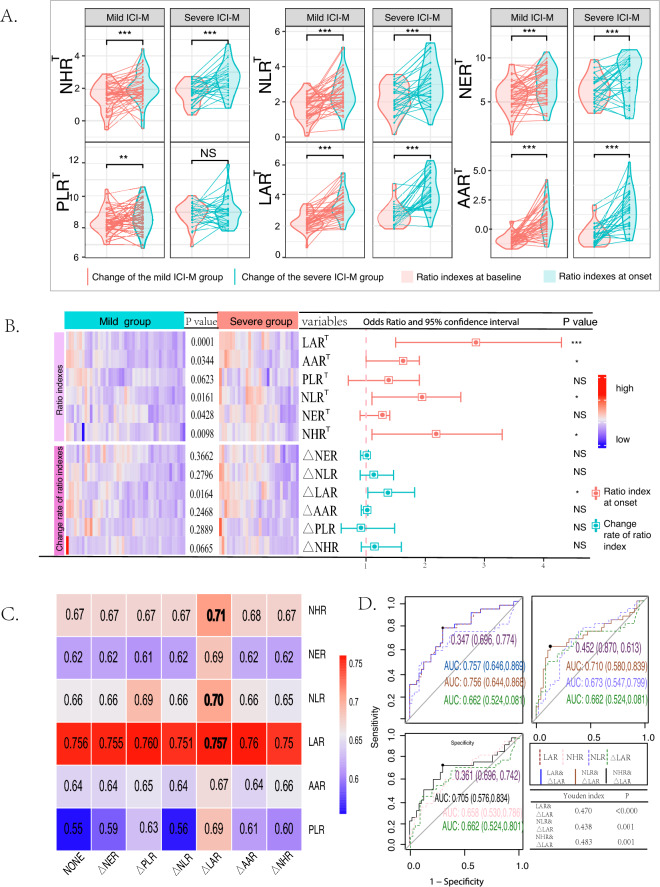
The diagnostic value of (the change rates of) ratio indexes for the severity of ICI-M. Comparison of ratio indexes at onset and their change rates by division of mild and severe groups (**A**), univariate logistic regression of (the change rates of)ratio indexes and their difference comparison between mild and severe groups after being standardized scaled (**B**), the AUC of the risk scores of different combination models and the ROC curves of the best performance models (**C** and **D**). *MACEs* Major adverse cardiovascular events; *ICI-M* Immune checkpoint inhibitors-related myocarditis; *AUC* Area under the curve; *ROC* Receiver operating characteristic; *NS* No significance; *P<0.01; **P<0.001; ***P<0.0001. $\phantom{0}^T$Logarithmic form of the ratio indexes to display

**Fig. 2:**
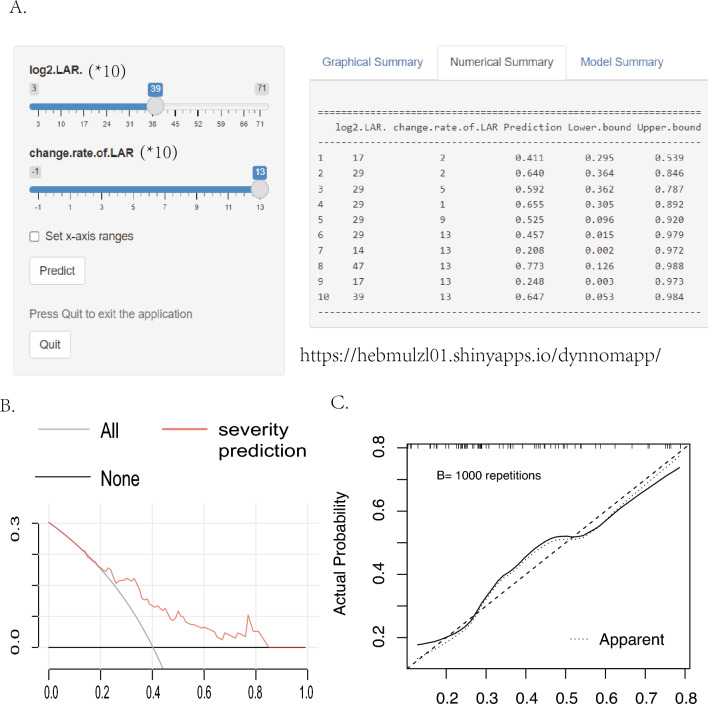
The dynamic nomogram based on $\textrm{LAR}^T$ and $\Delta$LAR for the prediction of severe ICI-M (**A**), decision curve analysis of the nomogram (**B**) and calibration curve for nomogram predictions of severe ICI-M survival by bootstrap with 1000 repetitions (**C**). *MACEs* Major adverse cardiovascular events; *ICI-M* Immune checkpoint inhibitors-related myocarditis. $\phantom{0}^T$Logarithmic form of the ratio indexes

Compared with baseline, the ratio biomarkers at onset were significantly elevated except PLR, when dividing the patients of ICI-M into the mild and severe groups (Fig. 1A). The ratio indexes at onset were higher significantly in severe ICI-M group than that in mild group except PLR; however, only the change rate of LAR displayed significantly higher tendency in the severe group when comparing among the two groups. In addition, univariate logistic analysis showed that NHR$\phantom{0}^T$, NLR$\phantom{0}^T$, LAR$\phantom{0}^T$, AAR$\phantom{0}^T$ at onset and $\Delta$LAR were all independently associated with severe ICI-M (Fig. 1B and Table S2). Furthermore, we sought to combine one initial ratio index with one change rate of the ratio index among the above indexes into bivariate logistic regression models and calculate the risk scores to process several ROC curve analysis to find the best one for the establish of nomogram-based model (Fig. 1C). Considering the significant factors of univariate logistic analysis, we finally selected the models containing these factors. Interestingly, when NLR$\phantom{0}^T$ [AUC, 95%CI 0.710 (0.580, 0.839) vs. 0.673 (0.547, 0.799)] and NHR$\phantom{0}^T$ [AUC, 95% CI 0.705 (0.576, 0.834) vs. 0.756 (0.644, 0.868)] combined with $\Delta$LAR respectively, the AUC improved significantly compared with themselves. However, LAR$\phantom{0}^T$ combined with $\Delta$LAR was selected to develop a predictive nomogram due to the best performance (Figs. 1D and 2A). The C-index of the prediction nomogram for severe ICI-M was 0.757 (95% CI: 0.646$-$0.869) and was 0.752 (95% CI: 0.635$-$0.866) after bootstrapping validation indicating good discrimination against the model. As displayed in Fig. 2, the calibration curve for severe ICI-M was very close to the actual result. The decision curve indicated that if the threshold probability of a patient or doctor was $>20$% and $<85$%, using this nomogram to predict the severity of ICI-M might add more benefits.

### The combination of NHR and LAR to predict the 40-day MACEs after ICI-M

**Fig. 3:**
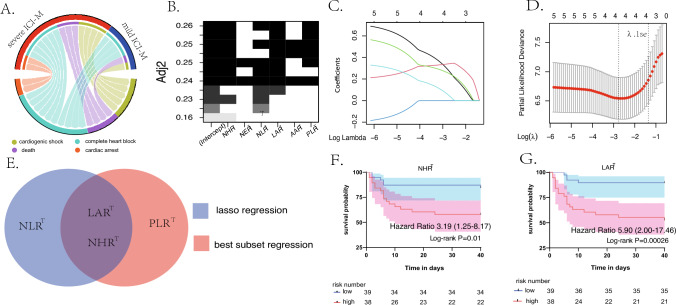
The proccesion of selecting the risk factors for MACEs after ICI-M. The composition of subsequent MACEs (**A**), Best subset regression (BSR) (**B**), least absolute shrinkage and selection operator (LASSO) regression analysis and tenfold cross-validation (**C** and **D**). Combination of two methods to select factors (**E**). Kaplan Meier curves of $\textrm{NHR}^T$ and $\textrm{LAR}^T$ seprated by the median (**F** and **G**). *MACEs* Major adverse cardiovascular events; *ICI-M* Immune checkpoint inhibitors-related myocarditis; *Adj2* Adjusted R-squared. $\phantom{0}^T$Logarithmic form of the ratio indexes

**Fig. 4:**
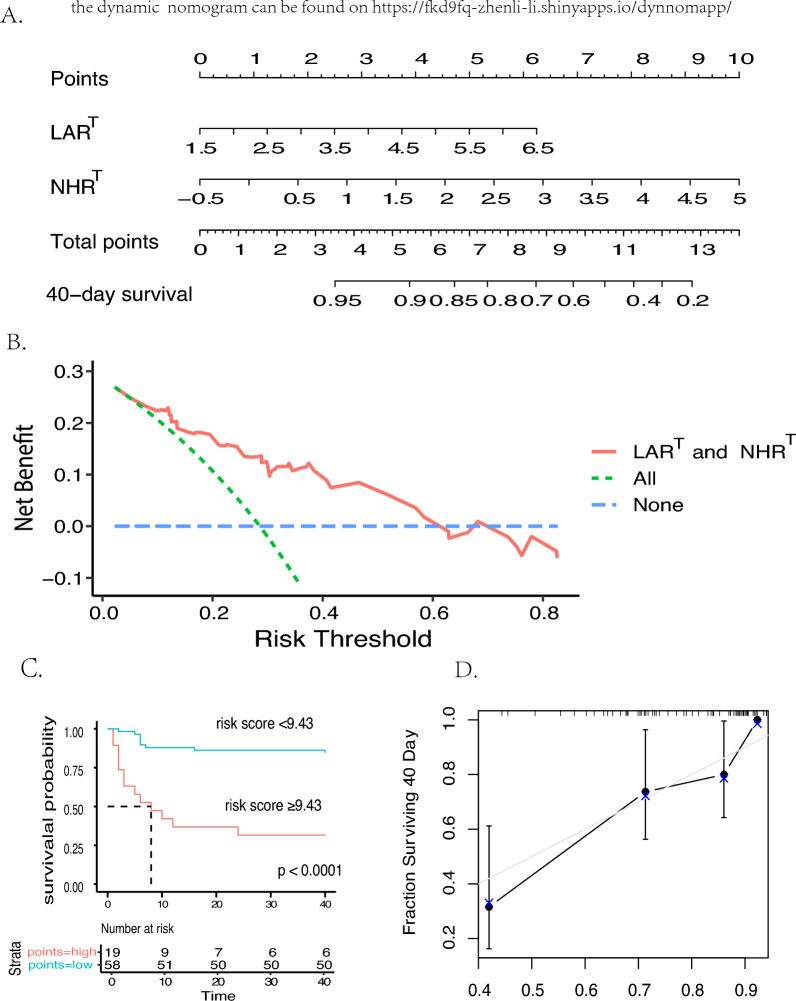
The nomogram based on $\textrm{NHR}^T$ and $\textrm{LAR}^T$ for the prediction of 40-day MACEs after ICI-M (**A**), decision curve analysis of the nomogram (**B**), the risk score $\ge$ 9.43 selected by “survminer” package performed best for risk stratification of 40-day MACEs survival (**C**) and calibration curve for nomogram predictions of 40-day MACEs survival by bootstrap with 1000 repetitions (**D**). *MACEs* Major adverse cardiovascular events; *ICI-M* Immune checkpoint inhibitors-related myocarditis. $\phantom{0}^T$Logarithmic form of the ratio indexes

The composition of MACEs is displayed in Fig. 3A. In the unicox regression and multicox regression by further adjustment of different confounders to find the reliable indexes associated with MACEs(Table S2), all ratio indexes displayed strong correlation with MACEs. Thus, we used two methods to select risk factors for further model construction. Firstly, 3 variables were selected with the highest adjusted R-squared in Best subset regression (BSR) analysis (Fig. 3B).Then, LASSO regression analysis with tenfold cross-validation identifed was performed on the ratio variables for MACEs prediction to prevent overfitting and solve severe collinearity problems; the result showed that 6 variables were reduced to 3 (lambda.1se = 0.024) (Fig. 3C and D). In the Kaplan-Meier curve analyses, NHR$\phantom{0}^T$ $\ge$ 2.05 and LAR$\phantom{0}^T$ $\ge$ 3.41 were signifcantly associated with the occurrence of subsequent MACEs after ICI-M (Fig. 3F and G). Considering the sample size of patients and the results of above methods, we finally selected $\textrm{NHR}^T$ and $\textrm{LAR}^T$ into the multicox regression for nomogram-based model construction, which also showed strong association in different multicox regression analyses(Fig. 4A and Table S4). The C-index for the prediction nomogram for 40-day MACEs was 0.807 (95% CI: 0.707$-$0.908) and was 0.779 (95% CI: 0.677$-$0.865) after bootstrapping validation indicating good discrimination against the model. As displayed in Fig. 4, the risk score $\ge$ 9.43 selected by “survminer” package performed best for risk stratification of 40-day MACEs survival. The calibration curve for 40-day MACEs showed good degree of fitting. The decision curve indicated that if the threshold probability of a patient or doctor was $>5$% and $<60$%, using this nomogram to predict the 40-day MACEs added more benefits.

### The association between change rates of ratio indexes and the MACEs after ICI-M

**Fig. 5:**
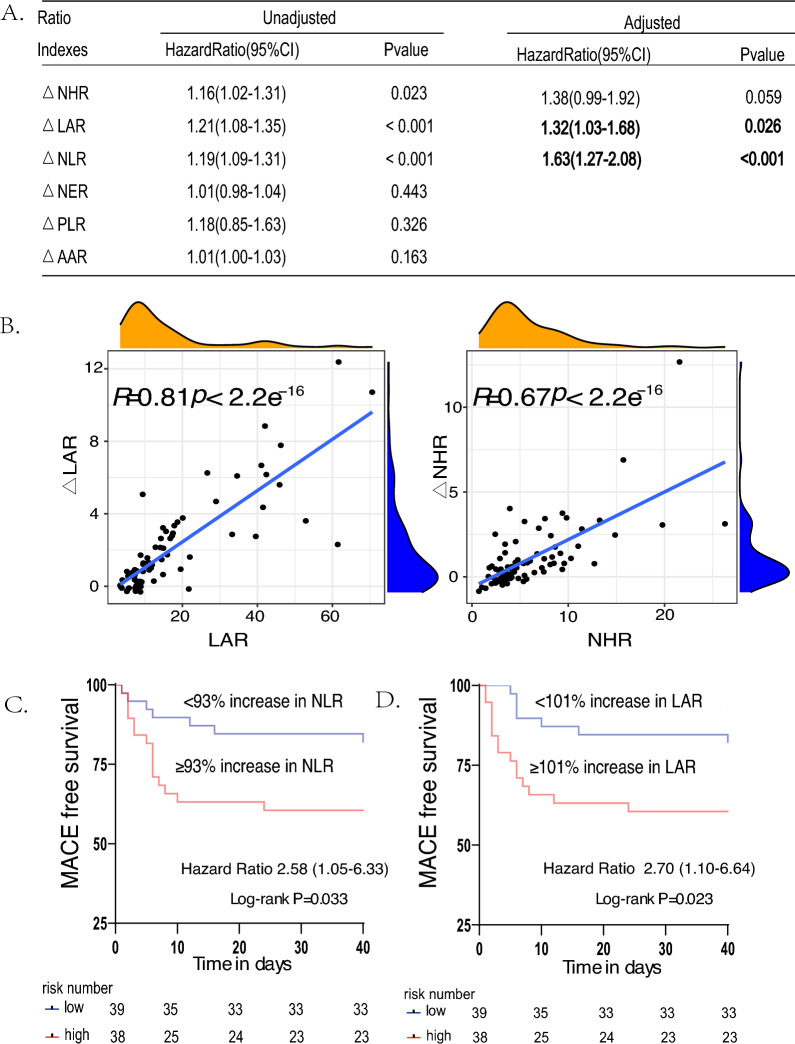
Unicox regression of the change rates of ratio indexes and multicox regression with further adjustment of basic information, tumor-related information, the immunotherapy-related factors and cardiac-related parameters (**A**), the Spearman correlation test of LAR (NHR) and $\triangle$LAR ($\triangle$NHR) (**B**) and Kaplan Meier curves of $\triangle$NHR and $\triangle$LAR seprated by the median (**C** and **D**).*MACEs* Major adverse cardiovascular events

We performed univariate Cox regression of the percentage change of the ratio biomarkers and it showed $\Delta$LAR, $\Delta$NLR and $\Delta$NHR are significantly associated with the subsequent MACEs after ICI-M. However, $\Delta$NHR lost its significance after further adjustment by different confounding factors (Fig. 5A). To explore the reason, we divided the patients into four groups by the severity of ICI-M and the occurrence of subsequent MACEs and performed paired Wilcoxon signed-rank test and Wilcoxon signed-rank test to compare difference. LAR at onset were significantly elevated from baseline in all groups. However, in the mild ICI-M group who develop MACEs, NHR (*p*=0.0625) showed no elevation tendency (Fig. S2A). Among the mild ICI-M group, the NHR of patients who developed MACEs was significantly higher than that of no-MACEs group. Nevertheless, such result lost significance in the severe group.Moreover, there was no difference between patients of severe ICI-M with or without MACEs and patients of mild ICI-M who developed MACEs (Fig. S2B). Then, the Spearman correlation analysis showed a positive correlation between LAR and $\Delta$LAR (r=0.81, *p*=2.2$\times e^{-16}$). A weaker correlation was shown between NHR and $\Delta$NHR (r=0.67, *p*=2.2$\times e^{-16}$) (Fig. 5B). Finally, we performed Kaplan-Meier curve analysis of $\Delta$LAR and $\Delta$NLR by using the median value to discriminate the ICI-M patients into two groups. A $\ge$ 93% increase in NLR and a $\ge$ 101% increase in LAR significantly separated patients in MACE-free survival (Fig. 5C and D).

## Discussion

### A summary of ratio indexes

Among ratio indexes, NLR, NER and PLR have been demonstrated to be indicators of diagnosis and prognosis in ICI-therapy and the mechanism of them has partly been explained (Takada et al. 2022; Afzal et al. 2019; Liang et al. 2023). However, some novel indexes remain to be exploited. Firstly, NHR can not only present both infammatory state and lipid metabolism but indicate the interaction between neutrophils and HDL-C, which is concerned with pathological processes of ACS by a mutually regulating role (Ren et al. 2023). Pan et al. (2023) demonstrated NHR was a useful indicator predicting cardiovascular risk for the early diagnosis of cardiovascular diseases. Secondly, neutrophils, known as the primary player in the acute infammatory response, assist the activation of monocytes and lymphocytes (Chakraborty et al. 2024). Cholesterol has been assessed as determinants of several alterations occurring in immune cells, which may enhance the activation of CD4+ and CD8+ T-cell (Pecci et al. 2024), which may result in a aggravated myocardial immune infiltration and a poor prognosis. Moreover, Pecci et al. (2024) also demonstrated the prognostic value of HDL-C for patients treated with ICIs. All the above explains NHR was a promising prognosis biomarker for cardiovascular prognosis after ICI-M.

As for LAR, LDH is a redox-active enzyme which is widely expressed across various tissues, primarily including the heart, skeletal muscle, liver, and brain, which is rapidly released into the peripheral blood after tissues are damaged (Peng et al. 2021). So higher LDH may reflect the inflammatory and injury level of myocardium among ICI-M patients at onset. Moreover, PKM2 plays a crucial role in mediating the metabolic transition to glycolysis following cardiac injury (Zeng et al. 2024), which may result in a acid environment of lactate in myocardium of ICI-M. A reduced expression of several glycolytic enzymes in CD4+ T cells induced by lactate makes these cells unable to egress from the inflamed tissue, which may promote atherosclerosis and myocardial inflammatory response (Certo et al. 2021; Dharmapuri et al. 2023). Serum ALB level not only reflects the nutritional status but represents a immune-related systemic inflammatory response, which is closely related to the prognosis of various tumors (Yan et al. 2021). A combination of LDH and ALB can reduce the disease induced bias and enhance the stability, which probably reflects a fatal and inflammatory state of heart. Similar to LAR, AAR has been proved to be associated with the prognosis of ICI-M, which is usually applied for liver diseases (Wu et al. 2023; Lai et al. 2024).

### The diagnostic value of ratio indexes onset to predict the severity of ICI-M

The mortality rate of ICI-M is high (38–46%), which calls for a crucial need for practical suspect, diagnose, and classification (Frascaro et al. 2023). Meanwhile, the National Comprehensive Cancer Network(NCCN) guidelines suggest the management of ICI-M according to severity from mild to severe by four grades stratification of diagnosis to perform suitable solutions immediately (Thompson et al. 2020). Thus far, Liang et al. (2023) have demonstrated NER at iRC onset can be involved in aid of staging iRCs severity. In addition, the change rates of ratio indexes from baseline to onset, to some extent, eliminates individual difference by a fusion of two time nodes and inherits a classification attribute. Several studies have exploited such change rates for clinical use. For example, $\Delta$NLR $>120\%$ was significantly associated with an increased risk for onset of grade3–4 irAEs (Takada et al. 2022). However, the ratio indexes at onset and their change rates from baseline to diagnose the ICI-M severity have not been fully exploited.

Based on these clues, we hypothesized the primary indexes and change rates of ratio indexes might have the ability to predict the severity of ICI-M. Meanwhile, our study exactly found these ratio indexes at onset, except PLR, were all significantly elevated but only LAR showed a different increase extent when dividing the ICI-M patients into the mild and the severe groups. The close correlation offered the probability to exploit effective ratio indexes to evaluate the severity of ICI-M. Interestingly, NHR, NLR and LAR separately combined with $\Delta$LAR performed better than single indexes in the evaluation. As for the improvement of distinction to ICI-M, a integration of inflammatory and damage indexes may better reflect the state of the myocardial tissue and decrease the discrepancy of reaction to ICI therapy of individuals. Our findings provided a method to assist diagnosing the severity of ICI-M, which supported the establishment of nomograms-based models in our study for further large-scale clinical practice.

### The ratio indexes at onset to predict the MACEs after ICI-M

The ability of LDH to reflect inflammation and damage degree makes LAR valuable. LDH has been demonstrated to be related to a bad prognosis in different cancer groups (Shiratori et al. 2023; Peng et al. 2021). In some studies, LDH was linked to the development of arrhythmia and ICU mortality after suffering a severe hit of myocardial cell (Lin et al. 2022) RN102; it was also described as a predictor of cardiac insufficiency at follow-up in elderly patients with acute myocardial infarction (Zhang et al. 2024). Moreover, many previous studies also found NHR was a unique indicator, which has been demonstrated to have a prognostic value of all-cause and cardiovascular mortality (Jiang et al. 2022); however, it has not been applied to ICI therapy so far.

Given all that, LAR and NHR may jointly contribute to the MACEs after ICI-M, which tend to represent a worse inflammatory environment and a damage load towards heart. Corresponding with the previous studies, our study highlighted the association of ratio biomarkers with the occurrence of subsequent MACEs after the ICI-M, where both NHR and LAR onset were significantly predictive in the multivariate Cox regression analyses. As we stated above, the combined effect of acid environment and active immune cells caused by lactate and cholesterol may exacerbate myocardial immuno-toxicity for poor cardiovascular outcomes. Recently, septal late gadolinium enhancement and reduced global longitudinal strain (GLS) was found to be a predictor of major cardiovascular events in patients with ICI-M (Awadalla et al. 2020; Cadour et al. 2022). Additionally, Mahmood et al. (2018) observed that a discharge level of cTnT $\ge$1.5 ng/mL was associated with an increase in the likelihood of a MACEs. In comparison, LAR and NHR are more financial, accessible and practical for clinical monitoring. To our best knowledge, we firstly applied LAR and NHR to the prediction of MACEs after ICI-M, which were able to discriminate short-term outcomes of ICI-M patients. Therefore, regular monitoring of changes in LAR and NHR could effectively identify the potential lethal events. However, the mechanism of inflammation induced by LAR and NHR reminds to be explored in fundamental experiment.

### The prognostic value of the change rates of ratio indexes for the MACEs after ICI-M

As we stated above, a higher change rates of ratio indexes probably represents a more fatal heart condition. Moreover, our study displayed a higer-tendency to develop MACEs among patients with higher change rates of part ratio biomarkers ($\Delta$LAR and $\Delta$NLR). In other studies, NLR was described as a significant long-term survival factor in different groups of patients with ICI therapy (Guven et al. 2022; Ota et al. 2020; Murakami et al. 2022). A recent study demonstrated a increase ($\ge$100%) in NLR was associated with subsequent MACEs after ICI-M, which are corresponded with our study. However, the studies about the $\Delta$LAR applied to the prognosis of MACEs prediction are rare. Our study firstly discovered a $\ge$101% increase in LAR may also function as a risk stratification tool for MACEs development as the performance of $\Delta$NLR.

Interestingly, $\Delta$NHR did not perform well as NHR to predict MACEs, but both LAR and $\Delta$LAR did. According to the analysis of our result, it may be partly explained that both LAR and $\Delta$LAR can directly reflect the degree of injuries of myocardium and immunotherapy reaction with a strong correlation due to the relatively stability of LAR before ICI therapy, as is similar to NLR (Liang et al. 2023). Conversely, NHR tends to be a cardiovascular risk factor highlighting both quantity threshold effect and varied ICI-therapy reaction due to different heart status, which depends on a instant higher-to-worse inflammatory mechanism in mild group and individual tolerance to inflammatory response in severe group. Hence, a high change rate of NHR may partly lose the predictive ability to the MACEs owing to bias within certain ICI-M groups. Nevertheless, further studies need to be carried out to verify our explanation.

### Clinical prospect and limitation

Recently, clinical prediction models have been exploit for assisting clinicians in predicting the diagnosis and prognosis of irAEs (Guo et al. 2024; Gao et al. 2023; Sung et al. 2023). However, the clinical prediction models for the severity and prognosis of ICI-M are still rare. To our best knowledge, we firstly established nomogram-based models to better manage the ICI-M for clinical practice, which showed good predictive accuracy and overall net benefit. Our models are of significant potential to guide clinicians in making appropriate decisions on risk stratification and optimizing patient care to some extent. Moreover, we have made dynamic nomograms and put them on https://hebmulzl01.shinyapps.io/dynnomapp and https://fkd9fq-zhenli-li.shinyapps.io/dynnomapp. To facilitate the external validation of our models for other clinical centers, we developed an online web page on https://hebmulzl.shinyapps.io/APPforexternalvalidition (Fig. S3), in which we just need to input a file in sav format according to the guidance and finish the validation procession. To better adapt to clinical demand in the future, we also developed an online web version of the APP on https://hebmulzl.shinyapps.io/nomogrambasedpredictivemoldel (Fig. S4).

Some limitations are as follows. Firstly, the low prevalence of ICIs-M and the retrospective nature of this study may limit the generalizability of our models. Hence, further external validation of larger and multi-center cohort is needed for our models. Therefore, we provided a tool online for further external validation to test the stability and effectiveness of our models for further clinical use. Secondly, endo-myocardial biopsy was not routine examination for the diagnosis of ICI-M in this study. Nevertheless, ICI-M cases were all confirmed by two cardiovascular specialists, which may reduce the bias of misdiagnosis. Additionally, the combination of ratio indexes and other circulating biomarkers should also be explored for the diagnosis and prognosis of ICI-M. Nevertheless, this study may provide a economical and practical choice for early diagnosis, detection and monitor for the severe ICI-M and the lethal MACEs.

## Conclusion

In this study, we found ratio indexes at onset and their change rates from baseline showed good diagnostic value for the severity of ICI-M and prognostic value for the subsequent MACEs, particularly LAR, NHR and their change rates. For further clinical practice, nomogram-based predictive models of ratio indexes were established and were with good performance after preliminary verification. This study would provide a potential choice for early detection and monitor of severe ICI-M as well as subsequent MACEs, if a large-scale, multicenter prospective validation could be completed. Our nomogram-based models may be useful in clinical practice as a simple and readily available prognostic tool.

## Supplementary Information

Below is the link to the electronic supplementary material.

Supplementary file 1 (pdf 876 KB)

## Data Availability

The datasets generated during the current study are available from the corresponding author on reasonable request.

## Abbreviations

LAR: Lactate dehydrogenase-to-albumin ratio
NHR: Neutrophil-to-high-density lipoprotein cholesterol ratio
NLR: Neutrophil-to-lymphocyte ratio
PLR: Platelet-to-lymphocyte ratio
NER: Neutrophil-to-eosinophil ratio
AAR: Aspartate aminotransferase-to-albumin ratio

## References

[CR1] Afzal MZ, Sarwar T, Shirai K (2019) Prognostic significance of hematological indices in malignant melanoma treated with immune checkpoint inhibitors. J Immunother 42(7):251–264. 10.1097/CJI.0000000000000272 31145229 10.1097/CJI.0000000000000272

[CR2] Awadalla M, Mahmood SS, Groarke JD et al (2020) Global longitudinal strain and cardiac events in patients with immune checkpoint inhibitor-related myocarditis. J Am Coll Cardiol 75(5):467–478. 10.1016/j.jacc.2019.11.049 32029128 10.1016/j.jacc.2019.11.049 PMC7067226

[CR3] Baik AH, Oluwole OO, Johnson DB, Shah N, Salem JE, Tsai KK, Moslehi JJ (2021) Mechanisms of cardiovascular toxicities associated with immunotherapies. Circ Res 128(11):1780–1801. 10.1161/CIRCRESAHA.120.315894 33934609 10.1161/CIRCRESAHA.120.315894 PMC8159878

[CR4] Cadour F, Cautela J, Rapacchi S, Varoquaux A, Habert P, Arnaud F, Jacquier A, Meilhac A, Paganelli F, Lalevee N, Scemama U, Thuny F (2022) Cardiac MRI features and prognostic value in immune checkpoint inhibitor-induced myocarditis. Radiology 303(3):512–521. 10.1148/radiol.211765 35230185 10.1148/radiol.211765

[CR5] Caforio AL, Pankuweit S, Arbustini E et al (2013) Current state of knowledge on aetiology, diagnosis, management, and therapy of myocarditis: a position statement of the european society of cardiology working group on myocardial and pericardial diseases. Eur Heart J 34(33):2636–2648. 10.1093/eurheartj/eht210. (**2648a-2648d**) 23824828 10.1093/eurheartj/eht210

[CR6] Certo M, Tsai CH, Pucino V, Ho PC, Mauro C (2021) Lactate modulation of immune responses in inflammatory versus tumour microenvironments. Nat Rev Immunol 21(3):151–161. 10.1038/s41577-020-0406-2 32839570 10.1038/s41577-020-0406-2

[CR7] Chakraborty C, Bhattacharya M, Lee SS (2024) Regulatory role of mirnas in the human immune and inflammatory response during the infection of sars-cov-2 and other respiratory viruses: A comprehensive review. Rev Med Virol 34(2):e2526. 10.1002/rmv.2526 38446531 10.1002/rmv.2526

[CR8] Dharmapuri S, Ozbek U, Jethra H et al (2023) Baseline neutrophil-lymphocyte ratio and platelet-lymphocyte ratio appear predictive of immune treatment related toxicity in hepatocellular carcinoma. World J Gastrointest Oncol 15(11):1900–1912. 10.4251/wjgo.v15.i11.1900 38077640 10.4251/wjgo.v15.i11.1900 PMC10701235

[CR9] Frascaro F, Bianchi N, Sanguettoli F, Marchini F, Meossi S, Zanarelli L, Tonet E, Serenelli M, Guardigli G, Campo G, Calabro L, Pavasini R (2023) Immune checkpoint inhibitors-associated myocarditis: diagnosis, treatment and current status on rechallenge. J Clin Med. 10.3390/jcm12247737 38137806 10.3390/jcm12247737 PMC10744238

[CR10] Gao W, Liu Q, Zhou Y, Yang M, Yu Y (2023) The predictive model construction for immune-related adverse events in non-small cell lung cancer patients receiving immunotherapy. Technol Cancer Res Treat 22:15330338231206704. 10.1177/15330338231206705 37927008 10.1177/15330338231206705 PMC10629333

[CR11] Guo J, Shu T, Zhang H, Huang N, Ren J, Lin L, Wu J, Wang Y, Huang Z, Bin J, Liao Y, Shi M, Liao W, Huang N (2024) A combined model of serum neutrophil extracellular traps, cd8(+) t cells, and tumor proportion score provides better prediction of pd-1 inhibitor efficacy in patients with nsclc. FEBS J. 10.1111/febs.17144 38661680 10.1111/febs.17144

[CR12] Guven DC, Sahin TK, Erul E, Cakir IY, Ucgul E, Yildirim HC, Aktepe OH, Erman M, Kilickap S, Aksoy S, Yalcin S (2022) The association between early changes in neutrophil-lymphocyte ratio and survival in patients treated with immunotherapy. J Clin Med. 10.3390/jcm11154523 35956139 10.3390/jcm11154523 PMC9369683

[CR13] Haj-Yehia E, Mincu RI, Korste S, Lampe L, Margraf SM, Michel L, Mahabadi AA, Ferdinandy P, Rassaf T, Totzeck M (2024) High neutrophil-to-lymphocyte ratio is associated with cancer therapy-related cardiovascular toxicity in high-risk cancer patients under immune checkpoint inhibitor therapy. Clin Res Cardiol 113(2):301–312. 10.1007/s00392-023-02327-9 37955712 10.1007/s00392-023-02327-9 PMC10850199

[CR14] Hu JR, Florido R, Lipson EJ, Naidoo J, Ardehali R, Tocchetti CG, Lyon AR, Padera RF, Johnson DB, Moslehi J (2019) Cardiovascular toxicities associated with immune checkpoint inhibitors. Cardiovasc Res 115(5):854–868. 10.1093/cvr/cvz026 30715219 10.1093/cvr/cvz026 PMC6452314

[CR15] Jiang M, Sun J, Zou H, Li M, Su Z, Sun W, Kong X (2022) Prognostic role of neutrophil to high-density lipoprotein cholesterol ratio for all-cause and cardiovascular mortality in the general population. Front Cardiovasc Med 9:807339. 10.3389/fcvm.2022.807339 35211525 10.3389/fcvm.2022.807339 PMC8861276

[CR16] Jo W, Won T, Daoud A, Cihakova D (2024) Immune checkpoint inhibitors associated cardiovascular immune-related adverse events. Front Immunol 15:1340373. 10.3389/fimmu.2024.1340373 38375475 10.3389/fimmu.2024.1340373 PMC10875074

[CR17] Lai X, Chen H, Dong X, Zhou G, Liang D, Xu F, Liu H, Luo Y, Liu H, Wan S (2024) Ast to alt ratio as a prospective risk predictor for liver cirrhosis in patients with chronic hbv infection. Eur J Gastroenterol Hepatol 36(3):338–344. 10.1097/MEG.0000000000002708 38251454 10.1097/MEG.0000000000002708 PMC10833202

[CR18] Liang L, Cui C, Lv D, Li Y, Huang L, Feng J, An T, Tian P, Yang K, Hu L, Gao L, Zhang J, Zhang Y, Ma F, Wang Y (2023) Inflammatory biomarkers in assessing severity and prognosis of immune checkpoint inhibitor-associated cardiotoxicity. ESC Heart Fail 10(3):1907–1918. 10.1002/ehf2.14340 36987542 10.1002/ehf2.14340 PMC10192297

[CR19] Lin L, Gao R, Chen L, Wu Z, Wei X, Xie Y (2022) Relationship between serum lactate dehydrogenase and mortality after cardiac arrest: a retrospective cohort study. Medicine (Baltimore) 101(45):e31499. 10.1097/MD.0000000000031499 36397356 10.1097/MD.0000000000031499 PMC9666175

[CR20] Liu G, Chen T, Zhang X, Hu B, Shi H (2024) Immune checkpoint inhibitor-associated cardiovascular toxicities: a review. Heliyon 10(5):e25747. 10.1016/j.heliyon.2024.e25747 38434280 10.1016/j.heliyon.2024.e25747 PMC10907684

[CR21] Mahmood SS, Fradley MG, Cohen JV et al (2018) Myocarditis in patients treated with immune checkpoint inhibitors. J Am Coll Cardiol 71(16):1755–1764. 10.1016/j.jacc.2018.02.037 29567210 10.1016/j.jacc.2018.02.037 PMC6196725

[CR22] Marzoog BA (2024) Early prognostic instrumental and laboratory biomarkers in post-mi. Cardiovasc Hematol Agents Med Chem. 10.2174/0118715257281715240108092557 38288831 10.2174/0118715257281715240108092557

[CR23] Moslehi J, Lichtman AH, Sharpe AH, Galluzzi L, Kitsis RN (2021) Immune checkpoint inhibitor-associated myocarditis: manifestations and mechanisms. J Clin Invest. 10.1172/JCI145186 33645548 10.1172/JCI145186 PMC7919710

[CR24] Murakami Y, Tamiya A, Taniguchi Y, Adachi Y, Enomoto T, Azuma K, Inagaki Y, Kouno S, Matsuda Y, Okishio K, Atagi S (2022) Retrospective analysis of long-term survival factors in patients with advanced non-small cell lung cancer treated with nivolumab. Thorac Cancer 13(4):593–601. 10.1111/1759-7714.14303 34989133 10.1111/1759-7714.14303 PMC8841702

[CR25] Ota Y, Takahari D, Suzuki T, Osumi H, Nakayama I, Oki A, Wakatsuki T, Ichimura T, Ogura M, Shinozaki E, Suenaga M, Chin K, Yamaguchi K (2020) Changes in the neutrophil-to-lymphocyte ratio during nivolumab monotherapy are associated with gastric cancer survival. Cancer Chemother Pharmacol 85(2):265–272. 10.1007/s00280-019-04023-w 31907646 10.1007/s00280-019-04023-w

[CR26] Pan X, Zhang X, Ban J, Yue L, Ren L, Chen S (2023) Association of neutrophil to high-density lipoprotein cholesterol ratio with cardiac ultrasound parameters and cardiovascular risk: A cross-sectional study based on healthy populations. J Inflamm Res 16:1853–1865. 10.2147/JIR.S406102 37138930 10.2147/JIR.S406102 PMC10150755

[CR27] Pecci F, Cantini L, Cognigni V et al (2024) Prognostic impact of blood lipid profile in patients with advanced solid tumors treated with immune checkpoint inhibitors: A multicenter cohort study. Oncologist 29(3):e372–e381. 10.1093/oncolo/oyad273 37796838 10.1093/oncolo/oyad273 PMC10911919

[CR28] Peng RR, Liang ZG, Chen KH, Li L, Qu S, Zhu XD (2021) Nomogram based on lactate dehydrogenase-to-albumin ratio (lar) and platelet-to-lymphocyte ratio (plr) for predicting survival in nasopharyngeal carcinoma. J Inflamm Res 14:4019–4033. 10.2147/JIR.S322475 34447260 10.2147/JIR.S322475 PMC8385134

[CR29] Ren H, Zhu B, Zhao Z, Li Y, Deng G, Wang Z, Ma B, Feng Y, Zhang Z, Zhao X, Ali Sheikh MS, Xia K (2023) Neutrophil to high-density lipoprotein cholesterol ratio as the risk mark in patients with type 2 diabetes combined with acute coronary syndrome: a cross-sectional study. Sci Rep 13(1):7836. 10.1038/s41598-023-35050-6 37188740 10.1038/s41598-023-35050-6 PMC10185574

[CR30] Shiratori F, Suzuki T, Yajima S, Oshima Y, Nanami T, Funahashi K, Shimada H (2023) Is high score of preoperative lactate dehydrogenase to albumin ratio predicting poor survivals in esophageal carcinoma patients? Ann Thorac Cardiovasc Surg 29(5):215–222. 10.5761/atcs.oa.23-00004 36858601 10.5761/atcs.oa.23-00004 PMC10587476

[CR31] Sung C, An J, Lee S et al (2023) Integrative analysis of risk factors for immune-related adverse events of checkpoint blockade therapy in cancer. Nat Cancer 4(6):844–859. 10.1038/s43018-023-00572-5 37308678 10.1038/s43018-023-00572-5

[CR32] Takada S, Murooka H, Tahatsu K, Yanase M, Umehara K, Hashishita H, Toru H, Satoru M, Sagawa T, Fujikawa K, Sato H, Mino K (2022) Identifying early predictive markers for immune-related adverse events in nivolumab-treated patients with renal cell carcinoma and gastric cancer. Asian Pac J Cancer Prev 23(2):695–701. 10.31557/APJCP.2022.23.2.695 35225483 10.31557/APJCP.2022.23.2.695 PMC9272606

[CR33] Thompson JA, Schneider BJ, Brahmer J et al (2020) Nccn guidelines insights: management of immunotherapy-related toxicities, version 1.2020. J Natl Compr Canc Netw 18(3):230–241. 10.6004/jnccn.2020.0012 32135517 10.6004/jnccn.2020.0012

[CR34] Vergara A, De Felice M, Cesaro A, Gragnano F, Pariggiano I, Golia E, De Pasquale A, Blasi E, Fimiani F, Monda E, Limongelli G, Calabro P (2023) Immune-checkpoint inhibitor-related myocarditis: where we are and where we will go. Angiology. 10.1177/00033197231201929 37699402 10.1177/00033197231201929

[CR35] Wu T, Zheng Z, Wang J, He M, Wang J, Pan Y, Chen J, Hu D, Zhang Y, Xu L, Chen M, Zhou Z (2023) Systemic inflammation score using pretherapeutic inflammatory markers to predict prognosis for hepatocellular carcinoma patients after hepatic arterial infusion chemotherapy. J Hepatocell Carcinoma 10:2133–2145. 10.2147/JHC.S437329 38058386 10.2147/JHC.S437329 PMC10697146

[CR36] Yan D, Huang Q, Dai C, Ren W, Chen S (2021) Lactic dehydrogenase to albumin ratio is associated with the risk of stroke-associated pneumonia in patients with acute ischemic stroke. Front Nutr 8:743216. 10.3389/fnut.2021.743216 34604286 10.3389/fnut.2021.743216 PMC8481374

[CR37] Yousif LI, Screever EM, Versluis D, Aboumsallem JP, Nierkens S, Manintveld OC, de Boer RA, Meijers WC (2023) Risk factors for immune checkpoint inhibitor-mediated cardiovascular toxicities. Curr Oncol Rep 25(7):753–763. 10.1007/s11912-023-01414-4 37079251 10.1007/s11912-023-01414-4 PMC10256640

[CR38] Zeng C, Wu J, Li J (2024) Pkm2: A potential regulator of cardiac injury through glycolytic and non-glycolytic pathways. J Cardiovasc Pharmacol. 10.1097/FJC.0000000000001568 38560918 10.1097/FJC.0000000000001568 PMC11230662

[CR39] Zhang L, Zlotoff DA, Awadalla M et al (2020) Major adverse cardiovascular events and the timing and dose of corticosteroids in immune checkpoint inhibitor-associated myocarditis. Circulation 141(24):2031–2034. 10.1161/CIRCULATIONAHA.119.044703 32539614 10.1161/CIRCULATIONAHA.119.044703 PMC7301778

[CR40] Zhang H, Kang K, Chen S, Su Q, Zhang W, Zeng L, Lin X, Peng F, Lin J, Chai D (2024) High serum lactate dehydrogenase as a predictor of cardiac insufficiency at follow-up in elderly patients with acute myocardial infarction. Arch Gerontol Geriatr 117:105253. 10.1016/j.archger.2023.105253 37956585 10.1016/j.archger.2023.105253

[CR41] Zhuang Y, An Q, Wang F, Han D, Qiao Z, Jiang Q, Liu M, Li Y, Shangguan J, Bi X, Shen D (2024) The role of circulating biomarkers in predicting the 30-day mortality of immune checkpoint inhibitors-related myocarditis: a retrospective cohort study. Intern Emerg Med 19(2):377–389. 10.1007/s11739-023-03481-8 38085435 10.1007/s11739-023-03481-8

