# Correction: Nomograms based on ratio indexes to predict severity and prognosis in immune checkpoint inhibitors-related myocarditis: a retrospective analysis

**DOI:** 10.1007/s00432-025-06110-3

**Published:** 2025-02-11

**Authors:** Zhenli Li, Tiezhu Yao, Guang Liu, Zhengkun Guan, Jing Liu, Ling Guo, Jingtao Ma

**Affiliations:** Department of Cardiology, The Fourth Hospital of Hebei Medical University, 12 Jiankang Road, Shijiazhuang, 050011 Hebei Provence People’s Republic of China

**Correction: Journal of Cancer Research and Clinical Oncology (2024) 150:277**

10.1007/s00432-024-05801-7

In this article, Tiezhu Yao, Guang Liu, Zhengkun Guan, Jing Liu, Ling Guo, and Jingtao Ma were originally denoted as equally contributing authors. The equal contribution statement has been removed at the request of all the authors.

The original article has been corrected.

