# Supplementary material for: Nomograms based on ratio indexes to predict severity and prognosis in immune checkpoint inhibitors-related myocarditis: a retrospective analysis
Source: J Cancer Res Clin Oncol. 2024 May 27;150(5):277. doi: 10.1007/s00432-024-05801-7 (PMC11602797; doi:10.1007/s00432-024-05801-7)

Title of the article	Ratio indexes as predictors of severity and prognosis in immune checkpoint inhibitors-related myocarditis: a retrospective analysis
Journal name	*Journal of Cancer Research and Clinical Oncology*
Author names	Zhenli Li, Tiezhu Yao, Guang Liu, Zhengkun Guan, Jing Liu, Ling Guo, Jingtao Ma
Affiliation and of the corresponding author	The Fourth Hospital of Hebei Medical University
E-mail address of the corresponding author	jingtaom0502@163.com
ORCID of the corresponding author	0009-0005-2427-4430

Supplementary material

Tables

Table S1 Laboratory test results and ratio indexes of patients with ICI-M at onset

Parameters	Total (N=77)	Severity of ICI-M: Mild group (N=46)	Severity of ICI-M: Severe group (N=31)	P value
**Circulating parameters**				
Neutrophils (10^9/L)	5.7 (3.8, 8.2)	4.6 (3.5, 6.9)	6.5 (5.6, 9.7)	0.007*
Lymphocyte (10^9/L)	0.91 ± 0.44	0.92 ± 0.41	0.89 ± 0.49	0.785
Eosinophil (10^9/L)	0.03 (0.01, 0.11)	0.04 (0.01, 0.12)	0.02 (0.01, 0.08)	0.285
Platelet (10^9/L)	202 ± 87	195 ± 73	213 ± 104	0.404
LDH (U/L)	378 (283, 638)	331 (264, 449)	638 (375, 1,163)	<0.001*
HDL-C(U/L)	1.19 (0.93, 1.45)	1.21 (0.90, 1.58)	1.13 (0.97, 1.30)	0.532
Albumin (g/L)	36.3(31.9, 41.9)	38.2 (34.0, 42.5)	34.5 (29.3, 36.7)	0.017*
AST (U/L)	67 (37, 208)	61 (37, 128)	117 (42, 263)	0.087
**Ratio indexes**				
LAR	11 (8, 18)	9 (7, 14)	16 (11, 39)	<0.001*
NHR	4.6 (3.2, 8.1)	4.0 (3.0, 5.7)	6.8 (4.3, 9.3)	0.010*
NLR	6 (4, 11)	5 (4, 9)	8 (5, 18)	0.019*
PLR	226 (148, 344)	199 (136, 343)	239 (164, 329)	0.334
NER	149 (48, 550)	141 (39, 373)	477 (82, 849)	0.041*
AAR	1.9 (1.1, 5.0)	1.5 (1.0, 3.3)	3.1 (1.3, 7.6)	0.039 *
**Cardiac-related parameters**				
Troponin I (ng/L)	120 (42, 766)	74 (22, 264)	510 (90, 1,850)	0.005*
NT-proBNP (pg/mL)	253 (123, 883)	157 (105, 590)	534 (230, 6,115)	0.008*
LVEF (%)	61 (58, 64)	62 (60, 65)	58 (53, 63)	0.006*

LDH Lactate dehydrogenase;*HDL-C* High-density lipoprotein;*AST* Aspartate aminotransferase;*LVEF* Left ventricular ejection fraction.

*Statistically signifcant association

Table S2 The univariate logistic regression of ratio indexes to predict the severity of ICI-M

Variables	Mild group	Severe group	Odds Ratio	95%CI	P value
**Initial indexes**					
NHR^T	2.1 ± 0.9	2.6 ± 0.9	1.97	(1.15-3.37)	0.014
NER^T	6.9 ± 1.9	7.7 ± 2.4	1.19	(0.95-1.48)	0.131
NLR^T	2.6 ± 1.0	3.3 ± 1.4	1.72	(1.11-2.64)	0.014
LAR^T	3.3 ± 0.9	4.2 ± 1.1	2.56	(1.51-4.36)	<0.001
AAR^T	0.9 ± 1.5	1.7 ± 1.7	1.40	(1.03-1.91)	0.034
PLR^T	7.7 ± 0.9	7.9 ± 1.1	1.22	(0.76-1.99)	0.411
**The change rates of ratio indexes**					
△NHR	0.9 ± 2.0	1.4 ± 1.7	1.16	(0.90-1.51)	0.253
△NER	4.9 ± 11.9	6.4 ± 11.4	1.01	(0.97-1.05)	0.575
△NLR	1.2 ± 1.6	2.2 ± 3.7	1.18	(0.96-1.45)	0.124
△LAR	1.3 ± 1.6	3.1 ± 3.3	1.38	(1.09-1.73)	0.007
△AAR	5.4 ± 9.4	9.4 ± 20.1	1.02	(0.98-1.06)	0.280
△PLR	0.5 ± 1.0	0.5 ± 1.3	0.95	(0.64-1.43)	0.820

ICI-M Immune checkpoint inhibitors-related myocarditis; *CI* Confidence interval.

^{T} Logarithmic form of the ratio indexes

Table S3 Unicox regression and and further adjustment of different confounders by multicox regression to find the reliable indexes associated with MACEs

Ratio	Unadjusted		Adjusted	
indexes	Hazard Ratio(95%CI)	P value	Hazard Ratio(95%CI)	P value
LAR^T	1.95 (1.34-2.82)	<0.001*	2.43 (1.29-4.59)	= 0.006*
AAR^T	1.42 (1.10-1.82)	= 0.006*	1.92 (1.15-3.19)	= 0.012*
NLR^T	2.03 (1.46-2.82)	<0.001*	3.75 (1.91-7.37)	<0.001*
NER^T	1.27 (1.02-1.59)	= 0.033*	1.42 (1.01-2.00)	= 0.045*
NHR^T	2.32 (1.48-3.64)	<0.001*	6.63 (2.22-19.85)	=0.001*
PLR^T	1.75 (1.13-2.73)	= 0.013*	5.28 (1.19-15.55)	= 0.004*

The confounders included factors of basic, tumor-related and immunotherapy-related information. *MACEs* Major adverse cardiovascular events; *CI* Confidence interval.

^{T}Logarithmic form of the ratio indexes

*Statistically signifcant association

Table S4 Multicox regression models of NHR^T and LAR^T with different confounders to predict the MACEs after ICI-M

Ratio	Model 1			Model 2			Model 3		
indexes	Hazard Ratio	95%CI	P value	Hazard Ratio	95%CI	P value	Hazard Ratio	95%CI	P value
NHR^T	4.68	2.04-10.69	<0.001*	4.54	1.91-10.77	0.001*	1.47	1.03-2.11	0.013*
LAR^T	1.99	1.26-3.15	0.003*	2.53	1.44-4.43	0.001*	1.21	1.08-1.35	< 0.001*

Model 1: Adjustment for age, sex, BMI, smoking history and comorbidities including hypertension, diabetes mellitus ,hyperlipidemia, stroke and pre-existing cardiovascular diseases; Model 2:Additional adjustment for tumor-related information; Model 3: Further adjustment for confounders including the immunotherapy-related factors and cardiac-related parameters. *MACEs* Major adverse cardiovascular events;*ICI-M* Immune checkpoint inhibitors-related myocarditis;*CI* Confidence interval.

^{T}Logarithmic form of the ratio indexes

*Statistically signifcant association

Figures

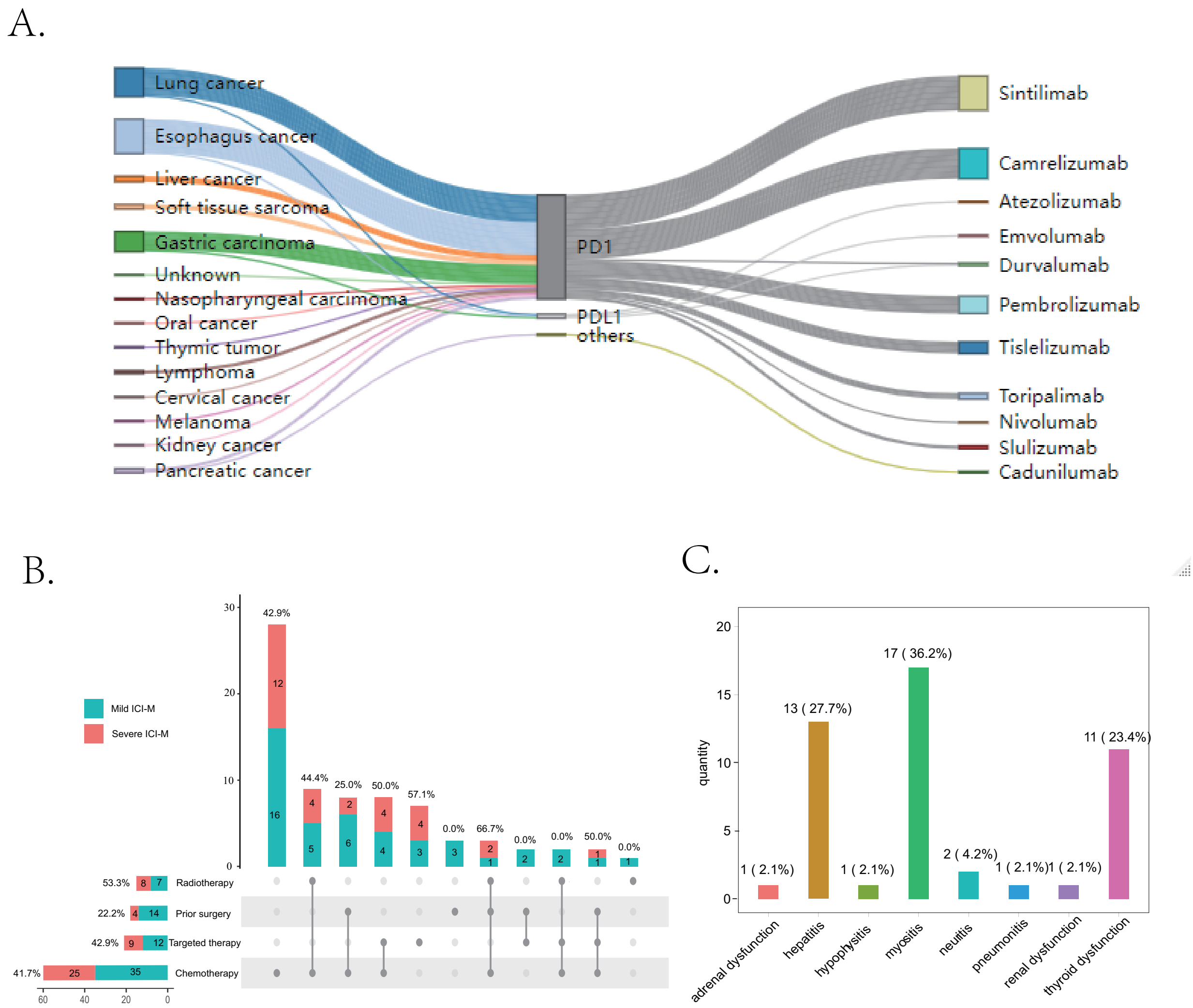

Fig. S1 The details of baseline information.The detailed administration of ICIs in different tumors(A), exact prior combined treatments among patients (B) and the composition of irAEs(C). *irAEs* Immune-related adverse events;*ICI-M* Immune checkpoint inhibitors-related myocarditis.

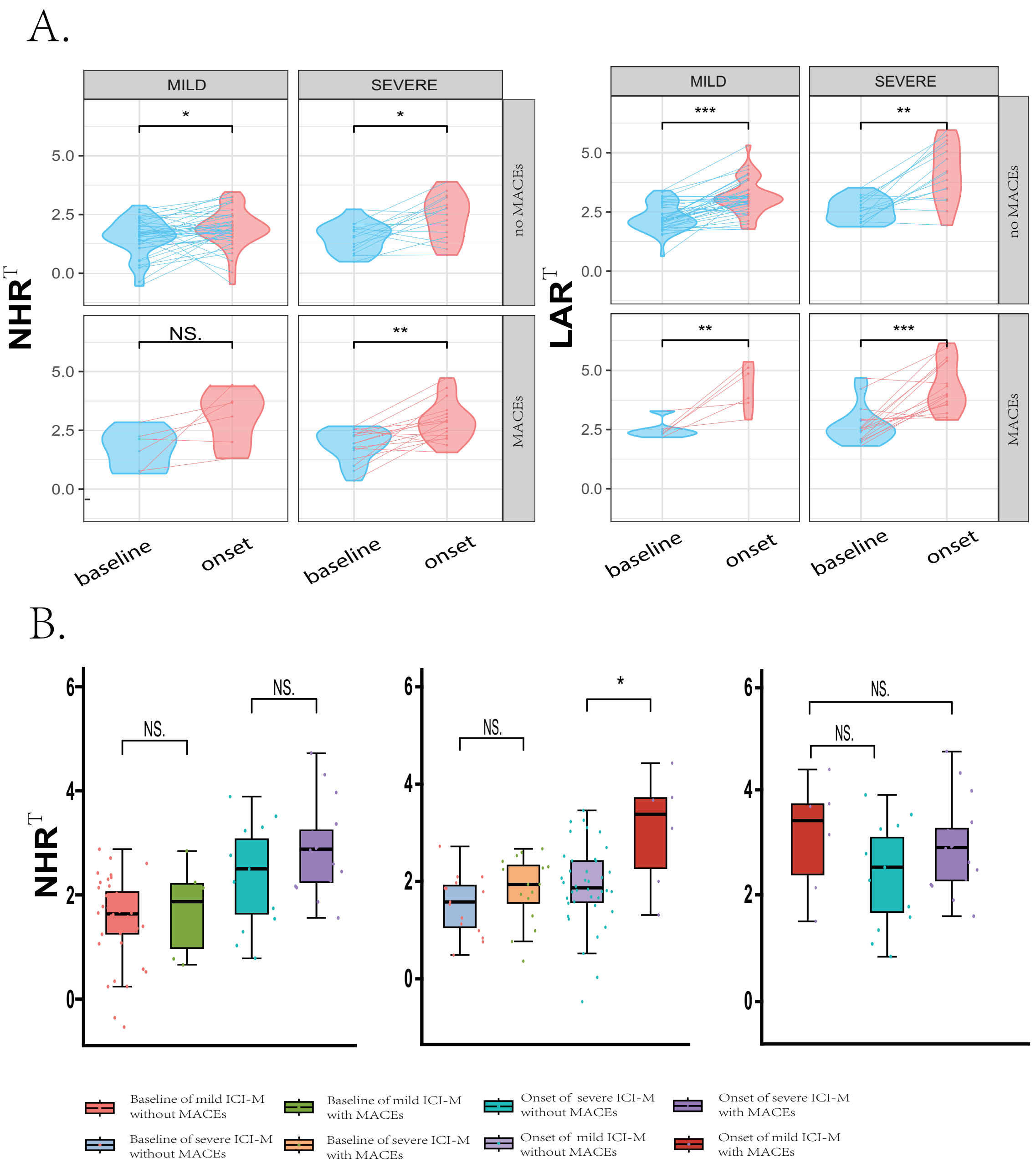

Fig. S2 The result of paired wilcoxon test and wilcoxon test when patients of ICI-M were divided into four groups by the severity of ICI-M and the occurrence of subsequent MACEs. *MACEs* Major adverse cardiovascular events; *ICI-M* Immune checkpoint inhibitors-related myocarditis.

^{T}Logarithmic form of the ratio indexes to display.

Fig. S3 Online app for external validation for nomogram based model and the operation interface.

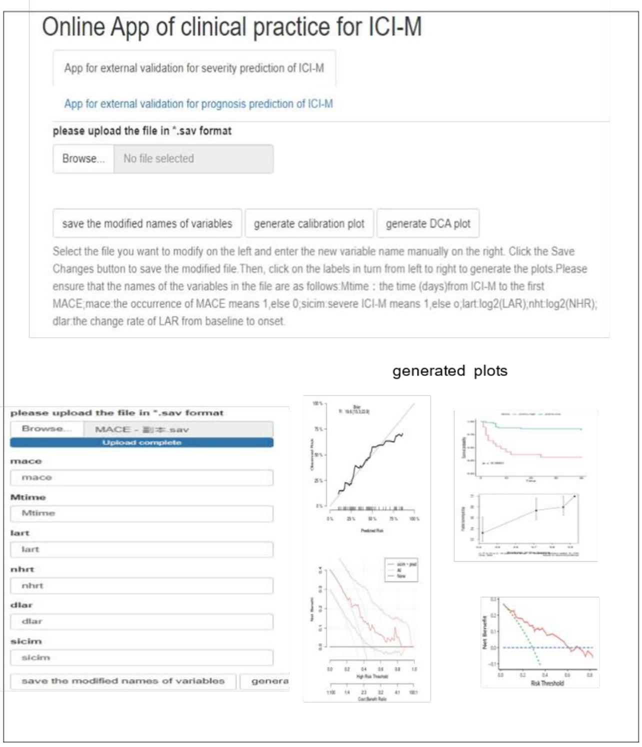

Fig. S4 Online app for future clinical use and the operation interface.

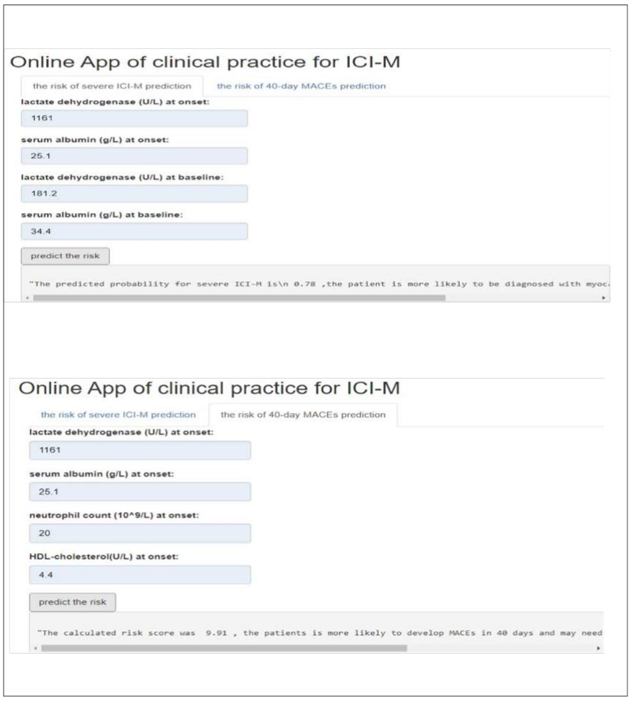

Supplement: Supplementary file 1 — (pdf 876 KB) [file 432_2024_5801_MOESM1_ESM.pdf]
